# Arabidopsis SFAR4 is a novel GDSL-type esterase involved in fatty acid degradation and glucose tolerance

**DOI:** 10.1186/s40529-015-0114-6

**Published:** 2015-12-01

**Authors:** Li-Min Huang, Chia-Ping Lai, Long-Fang O. Chen, Ming-Tsair Chan, Jei-Fu Shaw

**Affiliations:** 1 Institute of Biotechnology, National Cheng Kung University, No. 1, University Road, Tainan City, 701 Taiwan; 2 Department of Food and Beverage Management, Far East University, No. 49, Zhonghua Rd., Xinshi Dist., Tainan City, 74448 Taiwan; 3 Institute of Plant and Microbial Biology, Academia Sinica, No. 128, Sec. 2, Academia Road, Nankang, Taipei, 115 Taiwan; 4 Department of Biological Science and Technology, I-Shou University, No. 1, Sec. 1, Syuecheng Rd., Dashu District, Kaohsiung City, 84001 Taiwan; 5 Agriculture Biotechnology Research Center, Academia Sinica, No. 128, Sec. 2, Academia Road, Nankang, Taipei, 115 Taiwan; 6 Academia Sinica Biotechnology Center in Southern Taiwan, Academia Sinica, No. 59, Siraya Blvd., SinShih Dist., Tainan, 74145 Taiwan; 7 Agricultural Biotechnology Center, National Chung Hsing University, 250 Kuo Kuang Rd., Taichung, Taichung, 402 Taiwan

**Keywords:** SFAR, GDSL, Arabidopsis, Glucose tolerance

## Abstract

**Background:**

*SFARs* (*seed fatty acid reducers*) belonging to the GDSL lipases/esterases family have been reported to reduce fatty acid storage and composition in mature Arabidopsis seeds. GDSL lipases/esterases are hydrolytic enzymes that possess multifunctional properties, such as broad substrate specificity, regiospecificity, and stereoselectivity. Studies on the physiological functions and biochemical characteristics of GDSL lipases/esterases in plants are limited, so it is important to elucidate the molecular functions of GDSL-type genes.

**Results:**

We found that *SFAR4* (*At3g48460*), a fatty acid reducer belonging to the Arabidopsis GDSL lipases/esterases family, was intensely expressed in embryo protrusion, early seedlings, and pollen. The characterization of recombinant SFAR4 protein indicated that it has short-length *p*-nitrophenyl esterase activity. In addition, *SFAR4* enhanced the expression of genes involved in fatty acid metabolism during seed germination and seedling development. *SFAR4* elevated the expression of *COMATOSE*, which transports fatty acids into peroxisomes, and of *LACS6* and *LACS7*, which deliver long-chain acetyl-CoA for β-oxidation. Furthermore, *SFAR4* increased the transcription of *PED1* and *PNC1*, which function in importing peroxisomal ATP required for fatty acid degradation. *SFAR4* has another function on tolerance to high glucose concentrations but had no significant effects on the expression of the glucose sensor *HXK1*.

**Conclusions:**

The results demonstrated that SFAR4 is a GDSL-type esterase involved in fatty acid metabolism during post-germination and seedling development in *Arabidopsis*. We suggested that *SFAR4* plays an important role in fatty acid degradation, thus reducing the fatty acid content.

**Electronic supplementary material:**

The online version of this article (doi:10.1186/s40529-015-0114-6) contains supplementary material, which is available to authorized users.

## Background

GDSL-type esterases/lipases, a new subclass of lipolytic enzymes, are hydrolytic enzymes with multifunctional properties such as broad substrate specificity, regiospecificity, and stereoselectivity. They are widely distributed in bacteria and plants. GDSL lipolytic enzymes possess a distinct GDSL motif with a serine-containing active site near the N-terminus; hence, they differ from classical lipolytic enzymes with a G × S × G motif near the central conserved domain (Upton and Buckley 1995). Studies have illustrated that GDSL lipolytic enzymes have flexible activities, including thioesterase, protease, arylesterase, and lysophospholipase activities (Akoh et al. 2004; Molgaard et al. 2000; Li et al. 2000; Dalrymple et al. 1997). GDSL-type esterases/lipases have also been detected in various plant species, including *Arabidopsis*, rice, and maize, and they have been implicated in plant development, morphogenesis, and defense responses (Brick et al. 1995). Several GDSL lipase/esterases in various plant species possess substrate specificity. *BnSCE3*/*BnLIP2* from *Brassica napus* L. has been reported as a sinapine esterase that strongly expressed during seed germination and that functions in both germination and morphogenesis (Ling et al. 2006; Clauss et al. 2008, 2011). An acetyl-CoA carboxylase gene, *AmGDSH1*, was cloned in black-grass (*Alopecurus myosuroides*), and the characterization of the purified protein revealed that it was a carboxyesterase that activated the hydrolysis of the herbicide aryloxyphenoxypropionate (AOPP) (Cummins and Edwards 2004). Pepper *CaGLIP1* exhibited various activities, such as the hydrolysis of long-chain and short-chain *p*-nitrophenyl esters (Hong et al. 2008). A GDSL lipase/esterase isolated from sunflower (*Helianthus annuus* L.) seeds exhibited fatty acyl-ester hydrolase activity (Beisson et al. 1997). So far, plant GDSL-type esterases/lipases have not been proven to exhibit any lipase activity during seed germination or post-germinative growth or to mediate the relationship between stress and signaling pathway in *Arabidopsis*.

Gibberellic acid (GA) is essential for numerous aspects of plant development, such as seed germination, leaf expansion, stem elongation, flowering, and seed development (Richards et al. 2001; Sun and Gubler 2004). The DELLA proteins (RGA, GAI, RGL1, RGL2, and RGL3), comprising a class of GA signaling repressors, possess a highly conserved motif in their N-terminal domain in *Arabidopsis*. The presumptive DELLA-regulated genes involved in seed germination are highly distinct from those involved in flower development. An observation of the transcriptomes of DELLA mutants suggested that GA-mediated seed germination and flower development are distinctly controlled by DELLA-dependent transcriptomes (Cao et al. 2006). Recently, genes encoding several GDSL proteins, named *SFAR* genes, which are genes downregulated by DELLAs, have been reported to reduce fatty acid storage and composition in mature Arabidopsis seeds and to enhance tolerance to glucose stress during the seed germination (Chen et al. 2012). Among these genes, *SFAR4* has been demonstrated to reduce the total fatty acid content and change the composition of unsaturated fatty acids in storage seeds. Transgenic plants with overexpressed *SFAR4* exhibited tolerance to glucose stress and a higher germination rate (Chen et al. 2012). The relationship between glucose stress and *SFAR* genes in germinating seeds or during post-germinative seedling growth remains unknown. The mechanism underlying *SFAR4*-induced insensitivity to glucose in seeds or seedlings has not yet been elucidated.

Seed germination is associated with the degradation and mobilization of accumulated components in mature seeds (Bewley 1997). During germination, the Arabidopsis storage lipids are converted to sucrose through the glyoxylate cycle. Sucrose synthesis and the peroxisomal pathways are most crucial to post-germinative growth (Penfield et al. 2004; Holdsworth et al. 2008; Footitt et al. 2006). Sucrose stored in vacuoles or converted into glucose or UDP-glucose and fructose is essential as an energy source and for signaling during seed germination (Koch 2004). However, a high sugar concentration delays seed germination and inhibits cotyledon expansion and greening, true leaf formation, and root growth of Arabidopsis seedlings (Rolland et al. 2006). Exogenous glucose significantly retards the mobilization of seed storage lipids in germinating Arabidopsis seeds. Critical metabolic transition of the primary sugar source results in a loss of sensitivity to the inhibitory effect of glucose on lipid breakdown and early seedling development (To et al. 2002). Two genes involved in the glyoxylate cycle and playing a role in lipid metabolism, *malate synthase* (*MS*) and *isocitrate lyase* (*ICL*), have been reported to be negatively regulated by sugars. Using glyoxylate cycle mutants as a paradigms also reveals that they are resistant to the inhibitory effects of exogenous glucose on storage seed lipid breakdown in germinating seeds (Graham et al. 1994). Products of fatty acid catabolism can pass from the peroxisome to the mitochondrion independently of the glyoxylate cycle (Eastmond et al. 2000). Comprehensive metabolic profiling studies have suggested that Arabidopsis seed germination efficiency is not only affected by the accumulation of reserves during seed development or by their mobilization during seed germination but also by additional, as yet unknown factors (Fait et al. 2006).

Hayashi et al. used *ped* mutants, which are necessary to maintain glyoxysomal function, to demonstrate that peroxisomal fatty acid β-oxidation plays an important role in producing sucrose from storage lipids during germination (Hayashi et al. 1998). Studies on the deficiency of 3-ketoacyl-CoA thiolase, *PED1*, have suggested that peroxisomal β-oxidation is essential for post-germinative growth in *Arabidopsis* (Poirier et al. 1999; Hayashi et al. 1998). During post-germinative growth, peroxisomal β-oxidation of fatty acids releases storage triacylglycerol to produce acetyl-CoA and converts it to sucrose as a source of metabolic energy and carbon skeletons. Prior to β-oxidation, fatty acids are transported into peroxisomes by *CTS* (Footitt et al. 2002), and acyl-CoA substrates for β-oxidation are delivered by peroxisomal long-chain acyl-CoA synthetases (*LACS6* and *LACS7*) (Fulda et al. 2004). Both steps are required for post-germinative storage oil breakdown for successful seedling establishment. Recently, repressing peroxisomal adenine nucleotide carriers *PNC1* and *PNC2* were been found to impair peroxisomal ATP import, which inhibits fatty acid breakdown during early seedling growth (Linka et al. 2008). However, those studies indicate that β-oxidation of fatty acids is not required for Arabidopsis germination (Hayashi et al. 1998; Poirier et al. 1999; Fulda et al. 2004). Because fatty acids hydrolyzed from storage lipids are converted to CoA thioesters, storage lipid breakdown plays an important role during germination in oilseed plants (Graham and Eastmond 2002).

Exogenous sucrose delays lipid breakdown in Arabidopsis seedlings (Eastmond et al. 2000), and high sugar concentrations repress seed germination and seedling growth. However, the signal transduction pathways involved in responses to sugar are not fully known. Hexokinase (HXK) signaling may inhibit the mobilization of lipids and other storage compounds, thus preventing germination (Pego et al. 1999; Smeekens 2000). HXK and SNF1-related protein kinases are postulated to control several sugar-regulated processes. Hexokinase 1 (HXK1) is a sugar sensor with both signaling and metabolic functions in plants (Jang et al. 1997). In the sugar signaling pathway, HXK1 is a glucose sensor that modulates gene expression and multiple plant hormone-signaling pathways underlying sugar sensing and signaling in plants (Pego et al. 1999; Jang et al. 1997). Limited information exists regarding the role of GDSL-type genes in determining lipid metabolic cellular signaling during seed germination and post-germinative growth. It therefore becomes important to study the role of GDSL-type genes involved in lipid metabolic pathways and the mechanisms underlying sugar sensitivity.

This study provides evidence that *SFAR4* plays a crucial role in the lipid metabolic process and induction of tolerance to glucose stress through a glucose sensor-independent pathway during seed germination in *Arabidopsis*. Furthermore, the characterization of the recombinant SFAR4 reveals that it is a novel GDSL-type esterase. These observations suggest that *SFAR4* plays an important physiological role in seed germination, seedling growth, and susceptibility to sugar.

## Methods

### Sequence and motif analysis of SFAR4

Using the BLAST program to align the EST database for the *SFAR4* (*At3g48460*) sequence, full-length *SFAR4* cDNA was identified. Specific primers were designed to amplify the full-length *SFAR4* cDNA. The PCR products were cloned and sequenced. The sequence was subjected to BLAST analysis using the National Center for Biotechnology Information (NCBI) Arabidopsis genomic DNA database to identify the gene structure of *SFAR4*. The GDSL motif and catalytic triad sites of SFAR4 were characterized according to the SFAR4 sequence alignments to Arabidopsis GLIP1 and the typical GDSL-lipase-like enzymes within the MEGA 4.0 version software. The signal peptide and location of cleavage sites within the amino acid sequences were predicted using the SignalP 4.1 server. Motif analysis was performed with the Multiple Em for Motif Elicitation (MEME) motif search tool.

### Construction and transformation of SFAR4 in a yeast expression system

cDNA fragments of *SFAR4* were amplified and cloned into a pGAPZαC vector and expressed in *Pichia pastoris* SMD168H (Invitrogen). *P. pastoris* was cultured in YPD media (2 % peptone, 1 % yeast extract, 2 % glucose, and 1.5 % agar, pH 7.0). Plasmids were linearized with the restriction enzymes *Avr*II or *Bsp*HI and transformed into *P. pastoris* SMD168H by electroporation for 5 ms (Chang et al. 2006). High-voltage pulses (1.5 kV) were introduced to 100-µL samples in 0.2-cm electrode gap cuvettes using a BTX Electro Cell Manipulator apparatus. Transformed *P. pastoris* colonies of constructions were plated on YPD supplemented with zeocin (100 ng L^−1^, Invitrogen) for colony selection.

### Lipase or esterase activity plate assays, purification, and specific activity analysis

Recombinant SFAR4 of *P. pastoris* transformants were screened using a 1.5 % tributyrin emulsion agar or 2 % Tween-20 plates. The tributyrin solution was sonicated to a milky emulsion before adding the autoclaved YPD media. Tween-20 plates were prepared by using modified YPD media (YPD media supplemented with 0.5 % CaCl_2_, 2 % Tween-20, pH 7.0) (Cardenas et al. 2001). Lipase activity for tributyrin emulsion was determined by the appearance of clear rings around each colony, whereas for Tween-20, lipase activity was based on the formation of a white calcium precipitate. Colonies showing hydrolytic and lipolytic activity with respect to Tween-20 and tributyrin were isolated and further cultured and purified with HisTrap Ni Sepharose.

Enzyme activity was measured according to Chang et al. (2006). Lipase/esterase activity was assayed by using a spectrophotometer. The hydrolysis of *p*-nitrophenyl esters was conducted at 30 °C in 500 µL of 50 mM Good’s buffer (50 mM each of bicine, CAPS, sodium acetate, and bis–Tris propane), pH 7.0, containing 0.24 % Triton X-100 and 5 mM *p*-nitrophenyl esters. The increase in absorbance was recorded for 10 min at a wavelength of 348 nm. One unit of activity was defined as the quantity of enzyme necessary to release 1 µmol of *p*-nitrophenol per minute. Each experiment was conducted in triplicate. Error bars represented standard deviation (SD).

### Antibody preparation

For antibody preparation, the recombinant SFAR4 protein devoid of the putative signal peptide was amplified and cloned into a pET23a (+) vector and expressed in *E. coli* BL21 (DE3) competent cells (Novagen). The BL21 (DE3) transformants were cultured in LB media supplemented with ampicillin (50 ng L^−1^) at 37 °C overnight, then refreshed for 2 h and induced with 0.1 mM of IPTG for 6 h at 37 °C. The cultures were centrifuged to collect bacterial cells, which were then re-suspended in phosphate buffer and sonicated with a Misonix Sonicator XL2020. The sonication condition was 5 s on/off with 50–60 % amplitude on ice for 5 min with a 3-mm probe. Because of inclusion body production, the cell lysate was centrifuged at 20,000×*g* for 20 min at 4 °C and the pellet was collected. The pellet was washed twice with wash buffer (5 mM imidazole, 0.5 M NaCl, 20 mM phosphate buffer, pH 7.4 and 0.5 % Triton X-100), resuspended in buffer (8 M urea, 0.5 M NaCl, 20 mM phosphate buffer, pH 8.0), and homogenized with a tissue homogenizer. The supernatants following centrifugation (47,800×*g*, 20 min, 4 °C) were recovered and further purified. The soluble recombinant SFAR4s were purified through a HisTrap affinity column (GE Healthcare Life Sciences). The purified proteins were separated on the SDS–polyacrylamide gel (SDS-PAGE) and stained with Coomassie Brilliant Blue R-250 to confirm the molecular size and the purity. Purified recombinant SFAR4 protein was used for antiserum production. A rabbit polyclonal antibody specific to recombinant SFAR4 was produced by Allbio Science, Inc. (Taichung City, Taiwan), using a standard 70-day rabbit immunization protocol for rabbit polyclonal antibody production.

### Plant materials and growth conditions

To study the biological functions of *SFAR4* genes, we searched for putative *SFAR4* T-DNA insertional mutants from the T-DNA Express database of the SALK Institute Genome Analysis Laboratory (SIGnAL; http://signal.salk.edu/cgi-bin/tdnaexpress) and identified their exact integrated positions. Arabidopsis plants with ecotypes Col-0 were used as wild types for the phenotypic evaluation of plant growth and development. All of the Arabidopsis seeds underwent imbibition for 2 days and were then grown in soil at 22 °C under a 16-h light/8-h dark cycle. For aseptic growth, seeds were surface-sterilized and sown on Murashige and Skoog (MS) medium (Duchefa) and solidified with 0.7 % phytoagar (Duchefa). After sowing, the plates were incubated at 4 °C in the dark for 2 days and subsequently transferred to the growth chamber at 22 °C under 16-h light/8-h dark cycle. For osmotic and salt stress studies, Arabidopsis plants were surface-sterilized and then seeded on MS media plates supplemented with or without salts (NaCl, KCl, or LiCl), mannitol, and glucose at pH 5.7. Growth conditions were as described above. All data are the mean value of at least 50 plants, and these experiments were three replicates, obtaining similar values in each experiment. Data values of cotyledon expansion and greening for seedlings were statistically analyzed using the Student’s *t* test.

### Plant transformation

Transgenic plants were generated by *Agrobacterium* *tumefaciens*-mediated transformation in *Arabidopsis*. Full-length *SFAR4* was amplified and constructed into pCAMBIA1300-modified vectors that contained the constitutive cauliflower mosaic virus (CaMV) 35S promoter. Then, the constructs were transformed into *A. tumefaciens LBA4404* by electroporation. The transformants were screened with the *neo* gene (kanamycin-resistant) and introduced into plants by floral dip transformation (Clough and Bent 1998). The seeds from the transfected plants were harvested, surface-sterilized, and plated on a hygromycin selection media. Positive seedlings with 4 to 6 adult leaves were then transplanted and maintained in the greenhouse. Homozygous lines were identified in the next generation.

To study the expression pattern of *SFAR4*, *Pro*_*SFAR4*_:*GUS* transgenic plants with the β-glucuronidase (*GUS*) reporter gene under the control of the *SFAR4* promoter were cloned into the pBI101 plasmid, then generated by *Agrobacterium tumefaciens*-mediated genetic transformation as previously described. The length of *SFAR4* promoter is 2.1 kb upstream from the transcription start site. Col-0 wild-type plants were used for the construction of *Pro*_*SFAR4*_:*GUS* transgenic plants. An efficient and largely genotype-independent transformation of *Arabidopsis* was established based on the use of the *neo* gene as a selectable marker gene. The seeds from the transfected plants were harvested, surface-sterilized, and plated on the kanamycin selection media. Homozygous lines were identified in the next generation and the expression of GUS reporter gene was monitored by the GUS staining method. Staining for GUS activity was performed according to a modified protocol as Schoof et al. (2000) described. Plant material was prefixed in 90 % acetone for 20 min at room temperature, rinsed in staining buffer without 5-bromo-4-chloro-3-indolyl glucuronide (X-Gluc), and infiltrated with staining solution (50 mM NaPO_4_, pH 7.2; 2 mM potassium-ferrocyanide; 2 mM potassium-ferricyanide; 0.2 % Triton X-100; 2 mM X-Gluc) under vacuum on ice for 15 min and incubated at 37 °C for 3–8 h. After dehydration in an ethanol series up to 70 % EtOH, tissue was stored at room temperature until capturing the images. Photographs were taken with a Zeiss Microscope camera. To analyze the expression pattern of *SFAR4*, at least 10 independent lines of *Pro*_*SFAR4*_:*GUS* T_3_ homozygous were selected for the GUS staining experiments. In most cases, independent transgenic lines showed similar GUS activity patterns. However, examples of single lines that showed an expression pattern distinct from other lines were also identified. We reasoned that such lines may have the transgene inserted at a genomic site that aberrantly influenced expression or may have experienced a mutation upon transformation. Thus, GUS expression analyses are shown only when at least five independent transgenic lines displayed similar GUS expression patterns.

### Isolation of genomic DNA and genomic PCR and Southern blot

Correct T-DNA insertion lines were determined by using genomic PCR. Genomic DNA was isolated from individual plants as described by Winnepenninckx et al. (1995) and amplified by PCR reactions with a set of primers containing *SFAR4* gene-specific primers and a T-DNA left border specific primer (Additional file 1: Table S1). PCR products were sequenced and the accurate insertional site of T-DNA was confirmed. These mutants were then determined the T-DNA insertional number by Southern blot.

DNA blot was conducted according to the procedure of Solanas and Escrich (1997) with minor modifications and detected by the DIG-labeling detection system. 10 μg of genomic DNA was digested with a restriction enzyme and separated by 0.8 % agarose gel electrophoresis, and then transferred onto a nylon membrane. The membrane was incubated with a blocking solution for 1 h and then hybridized to a DIG-labeled single-stranded DNA probe overnight at 60 °C. Low stringency washes in 2× SSC/0.1 % SDS for 15 min at 37 °C were performed twice, followed by high stringency washes using 0.2× SSC/0.1 % SDS at 68 °C, following the DIG Application Manual from Roche (South San Francisco, CA, USA).

### RT-PCR, and quantitative real-time PCR and Statistical analysis

RNA was isolated from plants by using the RNeasy plant mini kit (Qiagen), and M-MLV reverse transcriptase was employed to generate cDNA. For the reverse transcription reactions, 2 µg of total RNA extracted from 14-day-old seedlings were incubated with 0.5 µg oligo dT in a total of 15 µL at 70 °C for 5 min. After chilling on ice for 5 min, components of the 1× RT buffer (Promega, Madison, WI, USA), 2.5 mM MgCl_2_, 0.5 mM dNTPs, 25 units of a ribonuclease inhibitor, and 200 units of Superscript RT were added as final concentrations and incubated at 42 °C for 60 min. After incubating at 70 °C for 15 min, four units of RNase H (Promega) were added and the reaction was incubated at 37 °C for 20 min. To examine the expression pattern of *SFAR4*, 1 μL of cDNA from the RT reactions and the specific primer pair for *SFAR4* were used to amplify the full-length cDNA of *SFAR4* by PCR.

Quantitative real-time RT-PCR was performed by the CFX Connect™ Real-Time PCR Detection System (Bio-Rad Inc.). Specific primers for each signal are shown in Additional file 1: Table S1. The melting curve was used to confirm the specificity of primers. Dissociation curves of each primer contained a single peak with no shoulders, and the agarose gels of the amplified product revealed single bands corresponding to the predicted amplicon length. All data are the mean value of three independent measures, and the error bars correspond to the standard error. Data values were statistically analyzed using the Student’s *t* test.

## Results and discussion

### Identification and characterization of *SFAR4*

*SFAR4*, as GDSL-like lipases/esterases, was localized on chromosome 3 and was comprised of 3 exons and 2 introns (Additional file 2: Fig. S1A). The full-length *SFAR4* cDNA was 1143 bp in length and encoded a 381-amino-acid protein (approximately 42.3 kDa). The GDSL motif and catalytic triad sites of the SFAR4 protein were characterized by aligning the SFAR4 sequence with the sequences of Arabidopsis GLIP1 and the typical GDSL-lipase-like enzymes using MEGA 4.0 version software. SFAR4 contained conserved motifs of typical GDSL lipase/esterase family proteins, which form the catalytic triad and oxyanion hole (Akoh et al. 2004). Four conserved residues, Ser-Gly-Asn-His, were present in the conserved blocks I, II, III, and V, respectively. Hence, SFAR4 is an SGNH-hydrolase (a subgroup of GDSL esterases/lipases) that contains a conserved GDSL motif near the N-terminus along with the predicted esterase/lipase catalytic triad active sites, Ser45, Asp187, and His347 (Additional file 2: Fig. S1B). The phylogenetic analysis of SFAR4 protein and 24 plant GDSL esterases/lipases (Additional file 3: Fig. S2) indicated that the evolutionary relationship of SFAR4 was homologous to that of BnSCE3/BnLIP2 in *Brassica napus,* myosuroides hydrolase (AmGDSH1) in the weed black-grass (*Alopecurus myosuroides*), acetylajmalan esterase (AAE) in Rauvolfia, and ARAB-1 in *Arabidopsis* (Ling et al. 2006; Cummins and Edwards 2004; Ruppert et al. 2005; Mikleusevic et al. 2009). We suggest that the molecular functions of *SFAR4* maybe contain the esterase activity but the physiological function is still unclear.

**Fig. 1:**
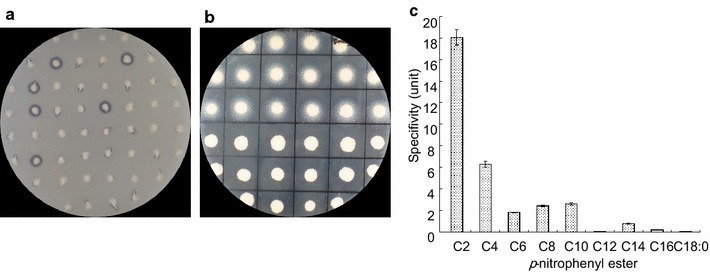
Recombinant SFAR4 proteins for lipase/esterase activity analysis. **a** YPD agar plates supplemented with 1.5 % tributyrin emulsion and secreted recombinant SFAR4 that hydrolyzes tributyrin to produce clear zones. **b** YPD supplemented with 0. 5 % CaCl_2_ and 2 % Tween-20, with hydrolytic activity indicated the formation of a white calcium precipitate. Transformed SFAR4 yeast colonies were grown on agar plates for 5 days. **c** Substrate specificity was determined by measuring activities for acyl *p*-nitrophenyl esters at 5 mM concentrations and monitoring the release of *p*-nitrophenol photometrically at 348 nm. These assays were performed using 50 mM Good’s buffer (pH 7.0) at 30 °C. One unit of enzyme is defined as the amount of enzyme that hydrolyzes 1 μmol of the substrate per minute

From the ontological annotation of molecular functions of the TAIR gene, SFAR4 is a secreted hydrolase acting on ester bonds, is involved in the glycerol biosynthetic and lipid metabolic processes, and is located in the endomembrane system (https://www.arabidopsis.org/servlets/TairObject?id=40278&type=locus). To characterize the specific activities of SFAR4, full-length *SFAR4* cDNA was cloned into an expression vector and then overexpressed in yeast (*Pichia pastoris*). However, analyses of the full-length recombinant SFAR4 protein revealed that it exhibited no esterase or lipase activity (data not shown). We presumed that SFAR4 might be processed or undergo post-translational modifications. Analysis of the full-length SFAR4 protein using the SignalP 4.1 server showed that SFAR4 possessed a signal peptide that targets the extracellular components (Additional file 2: Fig. S1). The signal peptide and location of cleavage sites in the amino acid sequences were predicted between residues 26 and 27 at the N-terminus (Additional file 2: Fig. S1B). The results indicated that the full-length *SFAR4* cDNA sequence encoded a precursor of SFAR4 with the signal peptide and that the precursor protein was further processed into a mature protein (355 amino acids) with a predicted molecular mass of 39.6 kDa. The *SFAR4* cDNA without a putative signal sequence was then cloned into the pGAPZαC vector (Invitrogen) and expressed in *P. pastoris*. The transformants with the recombinant SFAR4 protein without a putative signal peptide were selected by hydrolytic activity with Tween-20 and tributyrin (Fig. 1a, b). These transformants were further cultured and purified using HisTrap Ni Sepharose. The recombinant SFAR4 protein also showed esterase activity toward α-naphthyl butyrate (Additional file 4: Fig. S3) in native gel analysis. The specific activity of the purified enzyme was 6.25 µM min^−1^ mg^−1^ protein with *p*-nitrophenyl butyrate as a substrate and with approximately 1.75-fold purification (Table 1). The specific activity of the purified recombinant SFAR4 enzyme toward the *p*-nitrophenyl esters with 2- to 16-carbon acyl groups was analyzed. The results showed that SFAR4 protein is an esterase that favors esters with short acyl chains as substrates (Fig. 1c).

**Table 1:** Purification of enzymes catalyzing the *p*-nitrophenyl butyrate reaction from *Pichia pastoris*

Purified step	Total protein (mg)	Total activity (μM min^−1^)	Yield (%)	Specific activity (μM min^−1^ mg^−1^ protein)	Enrichment (fold)
Secreted medium (20 times concentrate)	2.00	7.16	100	3.58	1
HisTrap Sepharose	0.15	0.94	13.10	6.25	1.75

In this study, we found that recombinant SFAR4 protein expressed in *E. coli* was not active. However, the specific activity analysis of the recombinant SFAR4 protein expressed in yeast showed that it possessed hydrolase activity toward acyl esters (Fig. 1). The recombinant protein expressed in bacteria was deficient in post-translational modifications, whereas that expressed in *Pichia pastoris* underwent glycosylation modifications. The annotation in the UniPprot database (http://www.uniprot.org/uniprot/Q9STM6) predicted that SFAR4 possessed amino acid modifications in the form of glycosylation at residues 112, 140, and 158. Therefore, we suggested that SFAR4 proteins need post-translational modifications for their activities, such as amino acid glycosylation or correct folding.

### Expression pattern of *SFAR4*

The hormone GA plays a critical role in various processes of plant development, such as seed germination, leaf expansion, stem elongation, flowering, and seed development (Richards et al. 2001; Sun and Gubler 2004). It has been reported that DELLA proteins, which are GA signaling repressors, regulate the expression of numerous GDSL-type lipase/esterase genes during flower and seed development and germination (Cao et al. 2006). It has been demonstrated that five GDSL-type lipase/esterase genes, *SFAR1*-*5*, that were down-regulated by DELLA on DELLA mutant transcriptomes, are involved in reducing fatty acid storage and composition in mature Arabidopsis seeds (Chen et al. 2012). However, lipid hydrolysis is not essential for seed germination and seedling establishment (Kelly et al. 2011). Therefore, seed germination and development are distinctly controlled by DELLA-dependent transcriptomes. To analyze the diversity of DELLA-regulated GDSL-type lipase/esterase genes, the (MEME) program was used to elucidate the similarities among the conserved motifs of these proteins. Motifs among DELLA-downregulated GDSL-type enzymes with E-values from 1.02e−1356 to 9.9e−164 were found. Motifs 5, 9, 3, and 1 represent GDSL esterase/lipase conserved blocks I, II, III, and V, respectively. Most DELLA downregulated GDSL-type proteins with conserved motifs 6 and 7 differed significantly from SFAR4 (Additional file 5: Fig. S4). These different domains of SFAR4 may result in distinct activities or functions that are involved in the response of plant physiology to environmental changes.

**Fig. 2:**
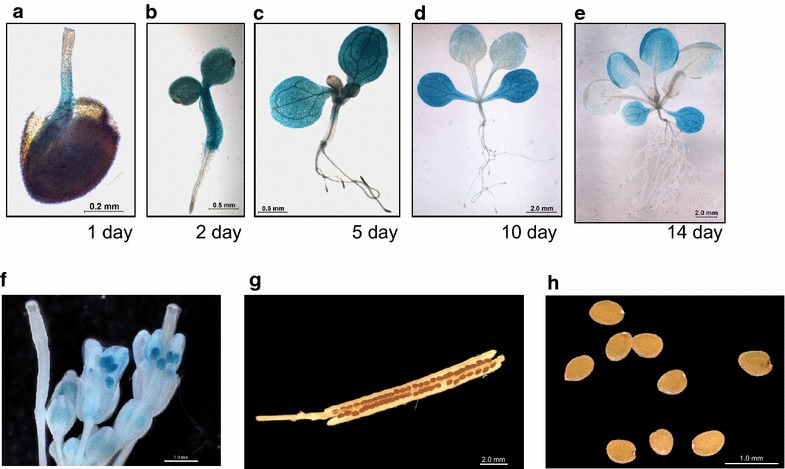
*SFAR4* promoters trigger *GUS* expression during the growth stage in *Arabidopsis*. **a**–**e** Shows 1–14 day-old *Pro*_*SFAR4*_:*GUS* transgenic seedlings. **f**, **g** and **h** represent flowers, mature siliques, and dry seeds, respectively. GUS expression in transgenic plants carrying *Pro*_*SFAR4*_:*GUS* is indicated in *blue* by staining for GUS activity

The sequencing study revealed that SFAR4 containing the classical motifs of GDSL-type lipases/esterases (Additional file 2: Fig. S1B), and a signal peptide may be targeted to the outside of the cell or to the membrane. The expression localization and stage of *SFAR4* are likely to play a role in specific functions during plant growth. To investigate the *SFAR4* expression pattern, transgenic plants carrying the *GUS* reporter gene under the control of the *SFAR4* promoter, including the region 2.1 kb upstream of the transcription start sites, were generated through *Agrobacterium tumefaciens*-mediated genetic transformation. The results of the *GUS* reporter gene assay with X-Gluc staining indicated that *SFAR4* is highly expressed during seed germination and early seedling growth (Fig. 2a–d). The expression of *GUS* in transgenic plants carrying *Pro*_*SFAR4*_:*GUS* was intense during embryo protrusion and in cotyledons, early rosette leaves, and pollen (Fig. 2f). *GUS* expression was low or undetectable in the apical meristem, roots, mature siliques, dry seeds, and floral regions except in the pollen (Fig. 2c–h). The results of *SFAR4* promoter triggers *GUS* gene expression were consistent with the observation of expression data collected from the Genevestigator microarray website (data not shown). These results indicate that the expression location and stage show the crucial role of *SFAR4* in seed germination and early seedling development. Specially, intense GUS reporter gene staining during embryo protrusion showed that *SFAR4* was highly expressed post-germination (Fig. 2a). These results indicate that *SFAR4* plays a crucial role in seed germination and seedling growth.

### SFAR overexpression enhanced the germination rate and young seedling establishment during high glucose stress

To study the biological functions of the *SFAR4* gene, we searched the Salk Institute database for putative T-DNA insertion mutants of *SFAR4* and obtained 2 alleles, *sfar4*-*1* (salk_122440) and *sfar4*-*2* (salk_008418), with T-DNA insertion in exon3 and exon1, respectively. From the SALK database, the T-DNA insertion sites in *sfar4*-*1* and *sfar4*-*2* were deduced to be in exon3 (1341 bp downstream of the ATG start codon) and exon1 (607 bp downstream of the ATG start codon). In this study, the results showed that the exact integrated sites were in exon3 (1567 bp downstream of the ATG start codon) and exon1 (605 bp downstream of the ATG start codon). The insertion site of salk_008418 is consistent with that deduced from the SALK database (Additional file 6: Fig. S5). A Southern blot analysis demonstrated that the *sfar4*-*1* and *sfar4*-*2* lines contained single T-DNA insertions, and RT-PCR revealed that the homozygous mutants of *sfar4*-*1* and *sfar4*-*2* harbored *SFAR4* transcripts that underwent gene silencing. To investigate the role of *SFAR4* genes in *Arabidopsis*, full-length cDNAs of transgenic plants expressing *SFAR4* under the control of a CaMV 35S promoter were generated through *Agrobacterium*-mediated transformation. The homozygous overexpressor and knockout mutant lines were confirmed using western blot analysis. The results indicated that the *SFAR4* overexpressors exhibited a higher *SFAR4* expression level than did the wild-type plants, whereas expression in the knockout mutants (*sfar4*-*1* and *sfar4*-*2*) was undetectable. The transgenic plants of two knockout mutants and overexpressors were further used to analyze the variant phenotypes (Additional file 6: Fig. S5).

**Fig. 3:**
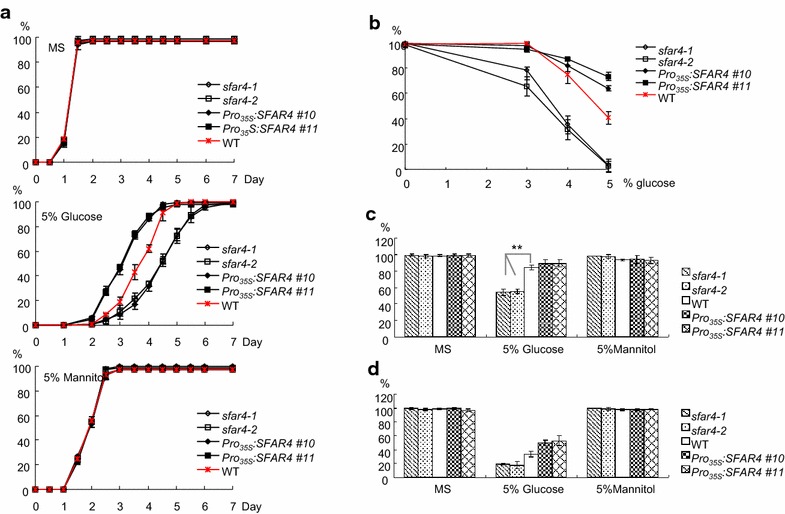
Phenotype analysis of *SFAR4* transgenic plants under osmotic (glucose and mannitol) stress. **a** Germination rates of *SFAR4* transgenic plants in MS medium with or without 5 % glucose or mannitol. *Solid lines* indicate *Pro*_*35S*_:*SFAR4* transgenic plants, whereas *hollow lines* indicate *sfar4* knockout mutants. Wild-type (Col-0) is indicated by *asterisks.* Germination was defined as complete protrusion of the radicle. **b** Germination rates of *SFAR4* transgenic plants in different concentrations of glucose at 84 h. **c**, **d** The cotyledon expansion and greening of *SFAR4* transgenic plants in MS medium plates with or without 5 % glucose or 5 % mannitol for 7 days. *sfar4*-*1* and *sfar4*-*2* represent knockout mutants, while *Pro*_*35S*_ *:SFAR4 #10* and *#11* indicate *SFAR4* overexpressors. WT indicates wild type (Col-0). The percentage of the cotyledon expansion and greening seedlings were measured. Each value is the mean of three independent measures, and the *error bars* correspond to the standard error (n = 50). *Asterisks* were used to indicate statistically significant difference compared with wild-type plants as determined by Student’s *t* test (*P < 0.05 and **P < 0.001, respectively)

The phenotypes of the *sfar4* knockout mutants and overexpressors with various plant growth and development characteristics were examined. The growth and morphology of leaves, seeds, siliques, and flowers in the transgenic plants were compared with those in the wild-type plants under normal growth conditions. However, the phenotypes of the knockout mutants, *sfar4*-*1* and *sfar4*-*2*, and the overexpressor plants, *Pro*_*35S*_ *:SFAR4#10* and *Pro*_*35S*_ *:SFAR4#11,* were similar to those of Col-0 wild-type plants. The phylogenetic analysis revealed that SFAR4 is closely related to these 24 GDSL-type proteins (Additional file 3: Fig. S2). According to the elucidated functions of these homologous genes (Additional file 7: Table S2), we suggested that *SFAR4* might be involved in the stress response. Therefore, we examined the responses of *sfar4* knockout mutants and *Pro*_*35S*_ *:SFAR4* transgenic plants under salt (LiCl, KCl, and NaCl) and osmotic (glucose and mannitol) stress. None of the mutants differed significantly from the wild-type plants in their germination and growth phenotypes on MS medium with or without salt during a 2-week observation period (Additional file 8: Fig. S6). Phenotype analysis of *SFAR4* transgenic plants under osmotic (glucose and mannitol) stress showed that the germination rate of *sfar4* knockout mutants was only 38 % after 4 days in MS medium with 5 % glucose (Fig. 3). By contrast, the germination rates in *SFAR4* overexpressors (*Pro*_*35S*_ *:SFAR4* transgenic lines) and Col-0 wild-type plants in 5 % glucose were 85 and 70 %, respectively (Fig. 3a). Both the expansion and greening of mutant cotyledons of *sfar4*-*1* and *sfar4*-*2* seedlings were more significantly inhibited by high glucose concentrations than those in wild-type seedlings (Fig. 3c). Compared with the wild-type plants, *sfar4*-*1* and *sfar4*-*2* were expanded only by approximately 55 % and green cotyledons by approximately 20 % in the presence of 5 % glucose. However, the exogenous supply of 5 % glucose affected cotyledon expansion in *Pro*_*35S*_ *:SFAR4* transgenic plants by approximately 90 % and in the Col-0 wild-type plants by approximately 85 %. This suggested that *SFAR4* disruption enhanced the susceptibility to glucose, whereas *SFAR4* overexpressors could partially mitigate this effect during seed germination. However, no statistically significant difference was observed between the transgenic and Col-0 wild-type plants during germination and cotyledon expansion or greening under mannitol treatment (Fig. 3a, c). These results suggest that hypersensitivity to glucose in the *sfar4* knockout mutants was not caused by osmotic response (Fig. 3; Additional file 8: Fig. S6).

### Expression of *HXK1* in *SFAR4* transgenic plants during seed germination

**Fig. 4:**
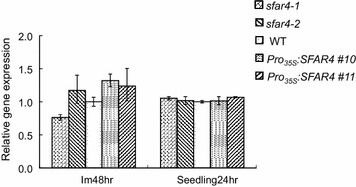
The relative transcription levels of *HXK1* in imbibed seeds (Im48hr) and post-germinated seedlings (24 h). The transcripts were determined by real-time PCR and normalized against actin. The transcription levels are relative to the WT set at level 1. *sfar4*-*1* and *sfar4*-*2* are knockout mutants; WT, wild type; *Pro*_*35S*_ *:SFAR4 #10* and *Pro*_*35S*_ *:SFAR4 #11* are *SFAR4* overexpressors. Each value is the mean of three independent measures, and the *error bars* correspond to the standard error. The statistical analysis of *HXK1* expression in wild type, *sfar4* knockout mutants and *SFAR4* overexpressors was performed by Student’s *t* test

According the results shown in Fig. 3, *SFAR4* overexpressors are insensitive to high glucose concentrations. These observations suggest that *SFAR4* in *Arabidopsis* encodes a functional protein that might play a key role in glucose susceptibility and glucose metabolic pathway regulation in germinating seeds. Arabidopsis *hexokinase 1* (*HXK1*) with the enzymatic function in glycolysis is suggested as a glucose sensor (Cho et al. 2006). A *hxk1* mutant known to be glucose insensitive2 (*gin2*-*1*) eliminated seedling developmental arrest induced by 6 % glucose (Moore et al. 2003), while *HXK1* overexpressors resulted in reduced hypocotyl elongation in seedlings and reduced plant growth (Karve et al. 2012). Hence we investigated that the relative transcription levels of *HXK1* in *SFAR4* transgenic plants compared with the WT (Col-0) imbibed seeds (Im48hr) and post-germinated seedlings (24 h). The statistical analysis for Student’s *t* test indicated that *HXK1* expressions have no significantly difference between wild type, *sfar4* knockout mutants and *SFAR4* overexpressors. These results indicated that *SFAR4* has no effect on *HXK1* expression (Fig. 4). Therefore, we suggest that SFAR4-meidated glucose response is HXK1-independent pathway.

### *SFAR* regulates downstream metabolic genes for peroxisomal β-oxidation

**Fig. 5:**
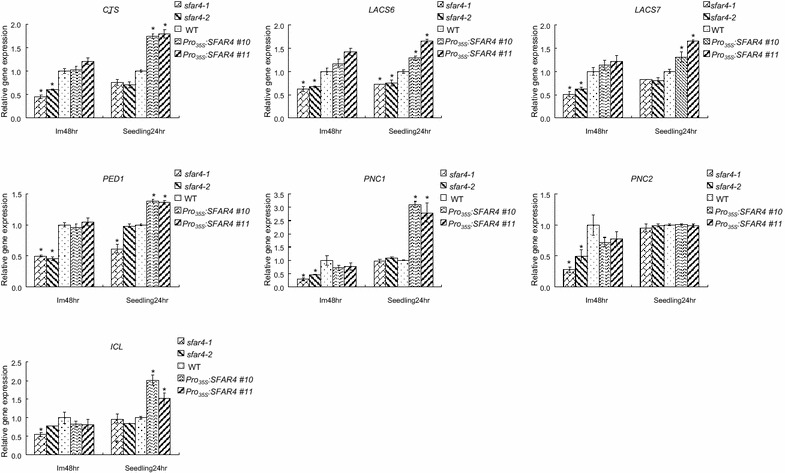
The relative transcription levels of *CTS*, *LACS6*, *LACS7*, *PED1*, *PNC1*, *PNC2* and *ICL* in imbibed (Im48hr) and post-germinated seedlings (24 h). The transcripts were determined by real-time PCR and normalized against the actin. For each gene, the transcription levels are relative to the WT (Col-0). *sfar4*-*1* and *sfar4*-*2* are knockout mutants; WT, wild type; *Pro*_*35S*_ *:SFAR4 #10* and *Pro*_*35S*_ *:SFAR4 #11* indicate *SFAR4* overexpressors, *Pro*_*35S*_ *:SFAR4* line 10 and 11. Each value is the mean of three independent measures, and the *error bars* correspond to the standard error. *Asterisks* indicate statistically significant difference compared with wild-type plants as determined by Student’s *t* test (*P < 0.05)

It has been reported that *SFAR4* reduces fatty acid storage and composition in developing seeds (Chen et al. 2012). However, how the gene works remains unclear. To investigate the cause of fatty acid reduction, we detected the expression of the regulatory genes that control fatty acid hydrolysis. The peroxisomal β-oxidation of fatty acids is a process that plays a crucial role in storage oil mobilization to support seedling establishment in oilseed plants, such as *Arabidopsis thaliana*. Fatty acids are metabolized through β-oxidation and converted to sucrose for supplying metabolic energy and carbon skeletons during post-germinative growth. Many mutants with a functional loss of β-oxidation that thereby diminishes seed germination or seedling growth have been studied. The *ped1* (peroxisome defective) mutant lacks thiolase activity required for β-oxidation. Because of weak fatty acid β-oxidation, *ped1* mutant plants cannot expand their leaves in the absence of sucrose as a result of reduced production of sucrose from storage lipids (Hayashi et al. 1998). *CTS* transports fatty acids into peroxisomes, and *LACS6* and *LACS7* deliver β-oxidation substrates (Footitt et al. 2002; Fulda et al. 2004). Both are required for storage oil breakdown for post-germinative seedling establishment. *CTS* regulates acyl CoA transport into the peroxisomes for β-oxidation and is a major control point for the switch between the opposing developmental programs of dormancy and germination (Footitt et al. 2002). To investigate the relationship between *SFAR4* and metabolic genes involved in β-oxidation, *CTS* and long-chain acyl-CoA synthetases, *LACS6* and *LACS7*, involved in β-oxidation were examined. *PED1* encoding a 3-ketoacyl-CoA thiolase required for β-oxidation was also examined. The results showed that *SFAR4* raised the expression levels of *CTS*, *LACS6*, *LACS7*, and *PED1* in imbibed and germinating seeds (Fig. 5). Suppressing importation of peroxisomal ATP inhibited fatty acid breakdown during early seedling growth. Repressing peroxisomal adenine nucleotide carriers, *PNC1* and *PNC2*, impaired fatty acid breakdown (Linka et al. 2008). We also detected *PNC1* and *PNC2* expression in wild-type and *SFAR4* transgenic plants in this study. The analysis of the transcription levels indicated that *SFAR4* upregulated *PNC1* but not *PNC2* during seedling development (Fig. 5). The β-oxidation of fatty acids produces acetyl-CoA, which is then converted to sucrose through the glyoxylate cycle and gluconeogenesis. The β-oxidation substrates were converted into sucrose through the dependent or independent glyoxylate cycle. The glyoxylate cycle is not critical, and an alternative mechanism exists that allows the utilization of fatty acids in plants. Therefore, the glyoxylate cycle is not essential for seed germination (Eastmond et al. 2000). However, it is considered essential for post-germinative growth and seedling establishment in oilseed plants (Eastmond et al. 2000). To elucidate the relationship between *SFAR4* and the glyoxylate cycle, *ICL*, the key factor of the glyoxylate cycle, was evaluated (Fig. 5). A comparison of *ICL* expression between the *Pro*_*35S*_ *:SFAR4* transgenic and wild-type plants indicated that *SFAR4* elevated *ICL* during the post-germination stage; however, no significant differences were observed in the imbibed seeds (Fig. 5). This study demonstrated that the gene expression of the glyoxylate cycle key enzyme *ICL* was regulated by *SFAR4* during embryo protrusion rather than in imbibed seeds (Fig. 5). We suggest that *SFAR4* reduced fatty acid content by up-regulating β-oxidation and the glyoxylate cycle during post-germinative growth and seedling establishment.

## Conclusions

In this study, we found that SFAR4 is a GDSL-type esterase that hydrolyzes short-length esters and suggested that SFAR4 requires post-translational modifications for attaining its enzymatic activity. This enzyme played a role in regulating fatty acid degradation through β-oxidation by up-regulating the expression of *CTS*, *LACS6*, *LACS7*, and *PNC1* but not *PNC2*. *SFAR4* overexpressors appear to induce tolerance to glucose during germination and seedling growth, and these studies suggest that *SFAR4* may play a crucial role in glucose-regulated pathways or glucose metabolism but not *HXK1*-dependent pathways.

## Additional files

**Additional file 1: Table S1.** Primers of PCR and RT-PCR.

**Additional file 2: Figure S1.** The genomic organization of *SFAR4,* and primary sequence and structure for SFAR4 protein. (A) Genomic organization of the *SFAR4* gene. (B) Protein sequence of SFAR4 deduced from the cDNA sequence. GDSL motif is indicated by the square. Catalytic triad sites Ser45, Asp187, and His347 are indicated by asterisks.

**Additional file 3: Figure S2.** Phylogenetic analysis of SFAR4 protein sequence and 24 plant GDSL lipase/esterases elucidated by Mega 4.0 with ClustalW and the NJ method with 1,000 bootstrap replicates. The nodes with less than 50% bootstrap support are not reported.

**Additional file 4: Figure S3.** The enzyme activity assay of recombinant SFAR4 proteins. (A) Recombinant SFAR4 proteins were expressed in *Pichia pastoris* (*SMD1168*). Secreted proteins were purified and separated by native PAGE and stained with Coomassie Blue. (B) The enzyme activity was determined by α-naphthyl butyrate. Lanes 1, 3, and 5 are the SFAR4 transformants. Lanes 2 and 4 are vector controls. Lanes 1 and 2 are the unconcentrated culture medium (20 μL/lane), while lanes 3 and 4 are concentrated solutions from the culture medium (10 μg/lane). Lane 5 shows purified recombinant SFAR4 proteins purified with HisTrap Ni Sepharose column from the concentrated solution (1 μg/lane).

**Additional file 5: Figure S4.** Protein motif structure and location of DELLA downregulated GDSL- type enzymes in *Arabidopsis*. Each colored box represents a particular motif. Their consensus sequence, accession numbers of DELLA regulated GDSL esterase/lipase proteins, and E-value are shown in the left frame. *At3G48460* is *SFAR4* as presented as a square box.

**Additional file 6: Figure S5.** Genotype analysis of *SFAR4* transgenic plants. (A) Genomic organization of *SFAR4* and location of the T-DNA insertion in *sfar4-1* and *sfar4-2*. The arrows indicate the positions of the T-DNA insertions (triangles). Genomic DNA of *SFAR4* is represented by 5′UTR, exons (black), introns (white) and 3′UTR. The T-DNA orientation of the left borders (LB) is indicated by the arrow. Chr3 refers to chromosome 3. (B) Southern blot analysis of the T-DNA insertion numbers in Col-0 and *sfar4* mutants. The genomic DNA was digested with *Hind*III, and the blot was probed with the T-DNA-specific DNA. (C) RNA analysis of *SFAR4* gene expression in *sfar4-1,* *sfar4-2,* and Col-0. *SFAR4* transcript expression was analyzed by RT-PCR. Total RNA (0.08 μg) was used to detect *SFAR4* and *Actin1* (loading control) expression. (D) SFAR4 proteins were detected by western blot with specific anti-SFAR4 antibody. RuBisCO was used as a loading control. The quantitative values were tested by Image J free software to analyze and quantify the intensity bands in western blot result images.

**Additional file 7: Table S2.** Accession numbers of 24 plant GDSL esterases/lipases.

**Additional file 8: Figure S6.** Phenotype of *SFAR4* transgenic plants. (A) Germination rates of *SFAR4* transgenic plants in MS medium with or without 10mM LiCl, 150mM KCl, or 200mM NaCl. Each value is the mean of three independent measures, and the error bars correspond to the standard error (n=50). (B) Images of Col-0 and *SFAR4* transgenic plants in MS medium with or without 10mM LiCl for 14 days. *sfar4-1* and *sfar4-2* are knockout mutants; WT, wild type; *Pro*_*35S*_ *:SFAR4 #10* and *Pro*_*35S*_ *:SFAR4 #11* indicate SFAR4 overexpressors, *Pro*_*35S*_ *:SFAR4* line 10 and 11. WT is wild type (Col-0).

## Abbreviations

GDSL: GDSL-motif
SFAR: seed fatty acid reducer
CTS: COMATOSE
HXK: hexokinase
GA: gibberellic acid
MS: malate synthase
ICL: isocitrate lyase
