# Supplementary material for: Arabidopsis SFAR4 is a novel GDSL-type esterase involved in fatty acid degradation and glucose tolerance
Source: Bot Stud. 2015 Dec 1;56:33. doi: 10.1186/s40529-015-0114-6 (PMC5432905; doi:10.1186/s40529-015-0114-6)
Supplement: Supplementary file 1 — Additional file 1: Table S1. Primers of PCR and RT-PCR. [file 40529_2015_114_MOESM1_ESM.docx]

**Supplementary Table S1:** Primers of PCR and RT-PCR

| LBa1 | 5′-TGGTTCACGTAGTGGGCCATCG-3′ |
| --- | --- |
| LBb1.3 | 5′-ATTTTGCCGATTTCGGAAC-3′ |
| SFAR4_F | 5′-ATGTCTTCCTCTATCTCTCCCCTC-3′ |
| SFAR4_R | 5′-TCATAAATAGTTTAGTTTCTTGATCAAC-3′ |
| Actin_F | 5′-GCTGATGGTGAAGACATTCAACCTC-3′ |
| Actin_R | 5′- GGTCACGACCAGCAAGATCAAGACG-3′ |
| 18s rRNA_F | 5′- GCTTGCTCTGATGATTCATG-3′ |
| 18s rRNA_R | 5′- TTGTCACTACCTCCCCGTGT- 3′ |
| CTS_F | 5′- AGAGTCCTTAGAGATATCTGGCCCA- 3′ |
| CTS_R | 5′- TGTATAAGGTCGCTGCGGGA- 3′ |
| PED1/KAT2_F | 5′- TGGTGTATTCAGGACATTTGC- 3′ |
| PED1/KAT2_R | 5′- TAAACCAGCCGCCTTAACTG- 3′ |
| LACS6_F | 5′- AAGAAGAACATCTTCAAATTGGCGC- 3′ |
| LACS6_R | 5′- TCACCATATATGAAGCACTGGCC- 3′ |
| LACS7_F | 5′- TCGGGTTGTGGTTACCTGGTG- 3′ |
| LACS7_R | 5′- CGTGAATGAAACACTGCGAAACG- 3′ |
| PNC1_F | 5′- CAGCAATCAGGTGCAAGG- 3′ |
| PNC1_R | 5′- TAAGCTTGGTGGTGGGAGTC- 3′ |
| PNC2_R | 5′- GGACTCTTGCGGAATGGTAA- 3′ |
| HXK1_F | 5′- AGATGCTGCTTTCTTTGGCGA- 3′ |
| HXK1_R | 5′- TTCAAGTCTGGAGAAGTGTCGTTGT- 3′ |
| ICL_F | 5′- AGATCGCAGACATCATCTGGATG- 3′ |
| ICL_R: | 5′- CTGCCAACAATATCCGAGCCT- 3′ |
