# Supplementary material for: Arabidopsis SFAR4 is a novel GDSL-type esterase involved in fatty acid degradation and glucose tolerance
Source: Bot Stud. 2015 Dec 1;56:33. doi: 10.1186/s40529-015-0114-6 (PMC5432905; doi:10.1186/s40529-015-0114-6)
Supplement: Supplementary file 2 — Additional file 2: Figure S1. The genomic organization of SFAR4, and primary sequence and structure for SFAR4 protein. (A) Genomic organization of the SFAR4 gene. (B) Protein sequence of SFAR4 deduced from the cDNA sequence. GDSL motif is indicated by the square. Catalytic triad sites Ser45, Asp187, and His347 are indicated by asterisks. [file 40529_2015_114_MOESM2_ESM.pdf]

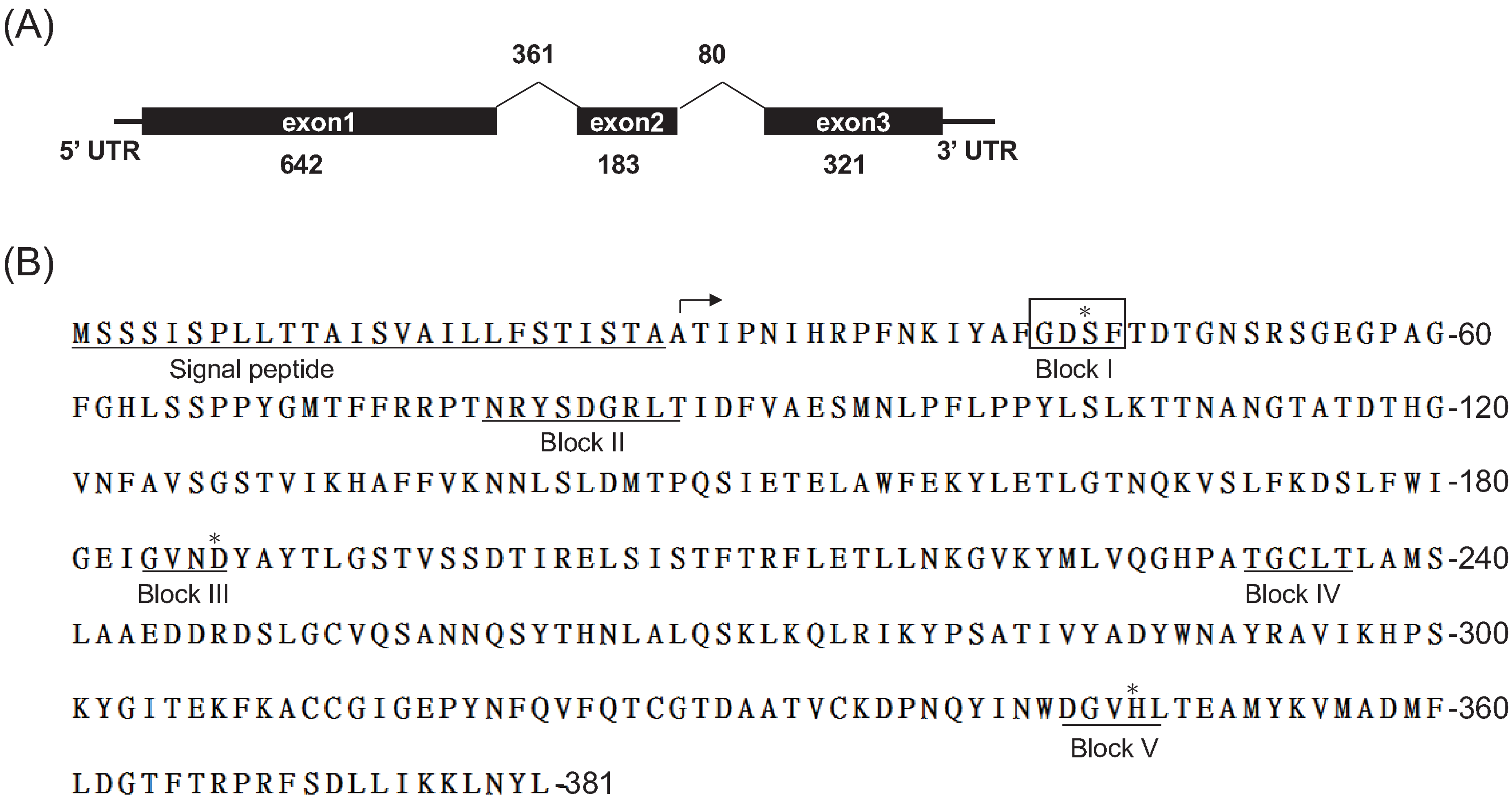

Supplementary Figure S1. The genomic organization of *SFAR4,* and primary sequence and structure for SFAR4 protein. (A) Genomic organization of the *SFAR4* gene. (B) Protein sequence of SFAR4 deduced from the cDNA sequence. GDSL motif is indicated by the square. Catalytic triad sites Ser45, Asp187, and His347 are indicated by asterisks.
