# Supplementary material for: Arabidopsis SFAR4 is a novel GDSL-type esterase involved in fatty acid degradation and glucose tolerance
Source: Bot Stud. 2015 Dec 1;56:33. doi: 10.1186/s40529-015-0114-6 (PMC5432905; doi:10.1186/s40529-015-0114-6)
Supplement: Supplementary file 3 — Additional file 3: Figure S2. Phylogenetic analysis of SFAR4 protein sequence and 24 plant GDSL lipase/esterases elucidated by Mega 4.0 with ClustalW and the NJ method with 1,000 bootstrap replicates. The nodes with less than 50% bootstrap support are not reported. [file 40529_2015_114_MOESM3_ESM.pdf]

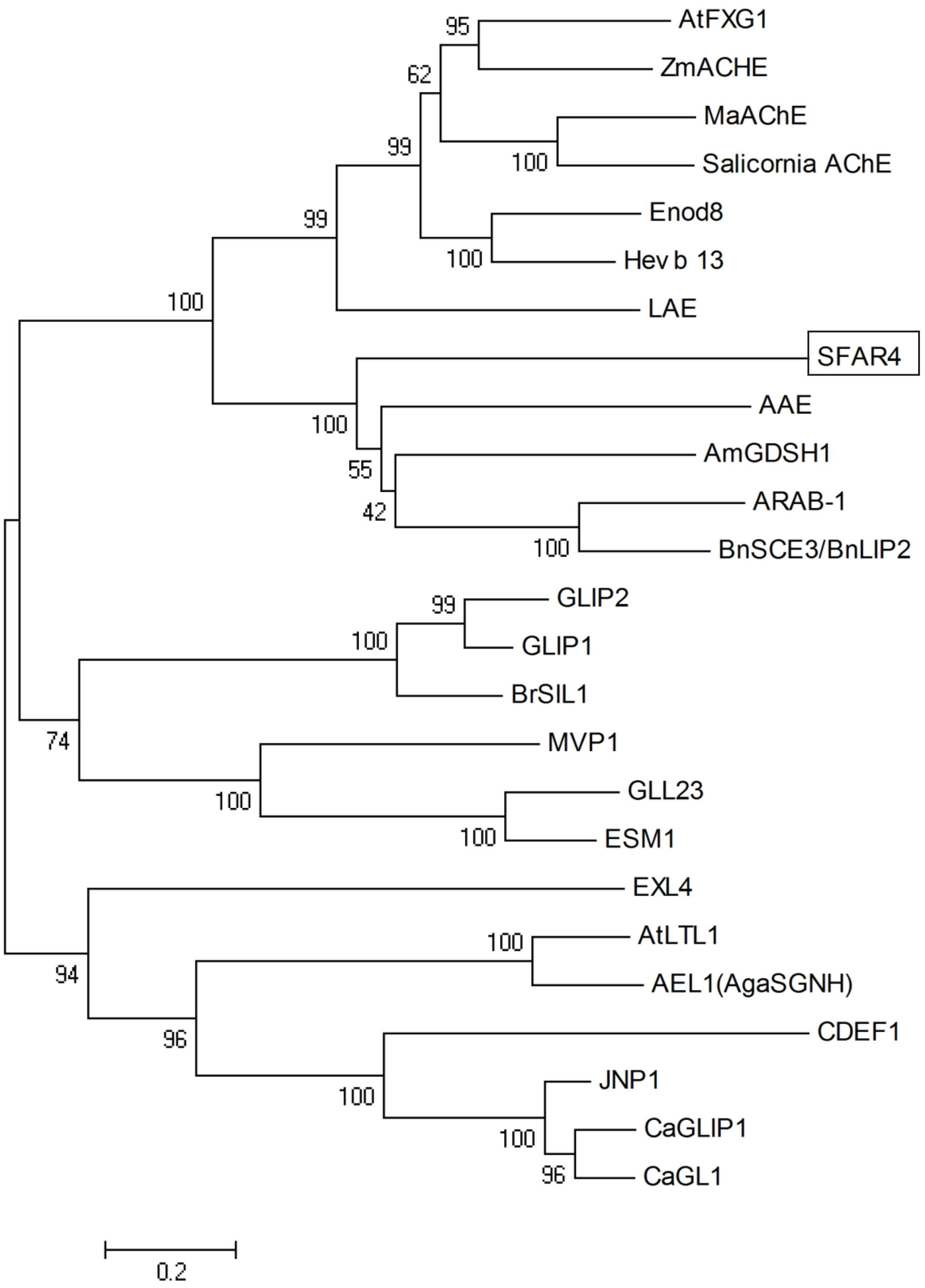

Supplementary Figure S2. Phylogenetic analysis of SFAR4 protein sequence and 24 plant GDSL lipase/esterases elucidated by Mega 4.0 with ClustalW and the NJ method with 1,000 bootstrap replicates. The nodes with less than 50% bootstrap support are not reported.
