# Supplementary material for: Arabidopsis SFAR4 is a novel GDSL-type esterase involved in fatty acid degradation and glucose tolerance
Source: Bot Stud. 2015 Dec 1;56:33. doi: 10.1186/s40529-015-0114-6 (PMC5432905; doi:10.1186/s40529-015-0114-6)
Supplement: Supplementary file 4 — Additional file 4: Figure S3. The enzyme activity assay of recombinant SFAR4 proteins. (A) Recombinant SFAR4 proteins were expressed in Pichia pastoris (SMD1168). Secreted proteins were purified and separated by native PAGE and stained with Coomassie Blue. (B) The enzyme activity was determined by α-naphthyl butyrate. Lanes 1, 3, and 5 are the SFAR4 transformants. Lanes 2 and 4 are vector controls. Lanes 1 and 2 are the unconcentrated culture medium (20 μL/lane), while lanes 3 and 4 are concentrated solutions from the culture medium (10 μg/lane). Lane 5 shows purified recombinant SFAR4 proteins purified with HisTrap Ni Sepharose column from the concentrated solution (1 μg/lane). [file 40529_2015_114_MOESM4_ESM.pdf]

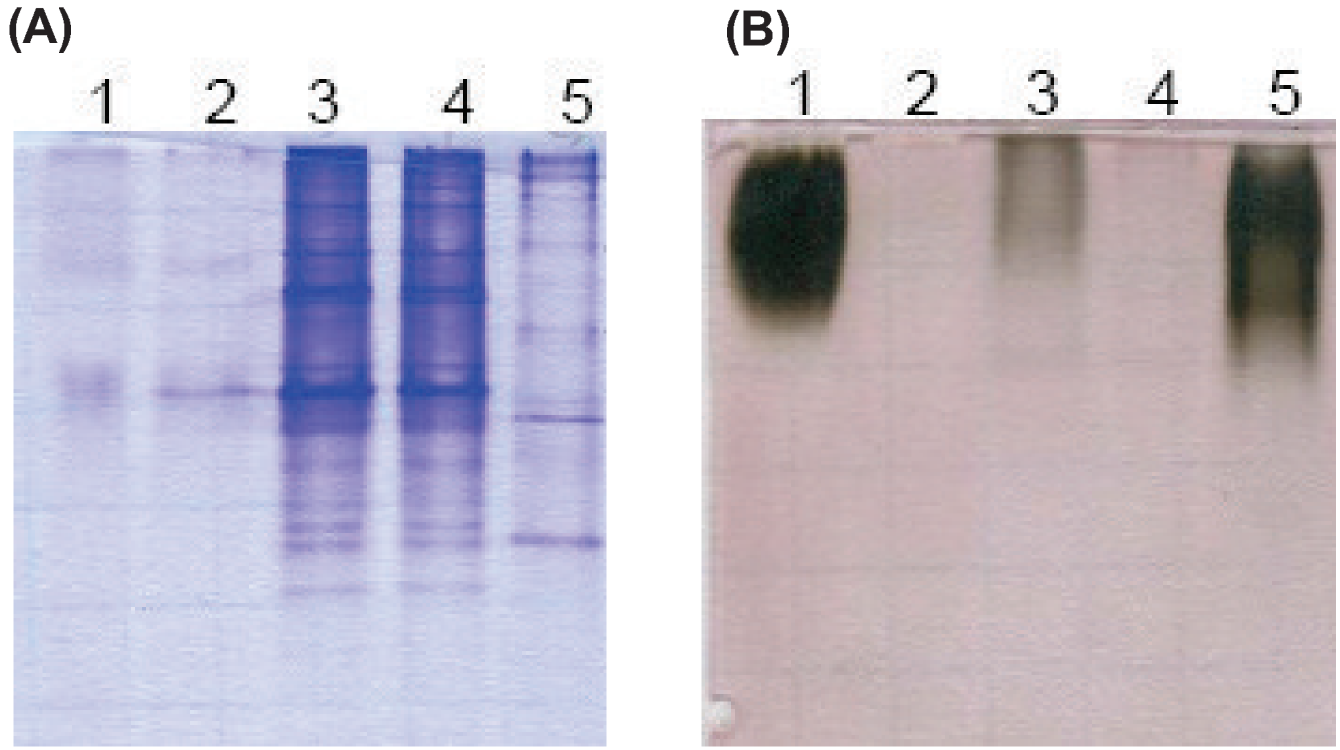


**Supplementary Figure S3.** The enzyme activity assay of recombinant SFAR4 proteins. (A) Recombinant SFAR4 proteins were expressed in *Pichia pastoris* (*SMD1168*). Secreted proteins were purified and separated by native PAGE and stained with Coomassie Blue. (B) The enzyme activity was determined by α-naphthyl butyrate. Lanes 1, 3, and 5 are the SFAR4 transformants. Lanes 2 and 4 are vector controls. Lanes 1 and 2 are the unconcentrated culture medium (20 μL/lane), while lanes 3 and 4 are concentrated solutions from the culture medium (10 μg/lane). Lane 5 shows purified recombinant SFAR4 proteins purified with HisTrap Ni Sepharose column from the concentrated solution (1 μg/lane).
