# Supplementary material for: Arabidopsis SFAR4 is a novel GDSL-type esterase involved in fatty acid degradation and glucose tolerance
Source: Bot Stud. 2015 Dec 1;56:33. doi: 10.1186/s40529-015-0114-6 (PMC5432905; doi:10.1186/s40529-015-0114-6)
Supplement: Supplementary file 5 — Additional file 5: Figure S4. Protein motif structure and location of DELLA downregulated GDSL- type enzymes in Arabidopsis. Each colored box represents a particular motif. Their consensus sequence, accession numbers of DELLA regulated GDSL esterase/lipase proteins, and E-value are shown in the left frame. At3G48460 is SFAR4 as presented as a square box. [file 40529_2015_114_MOESM5_ESM.pdf]

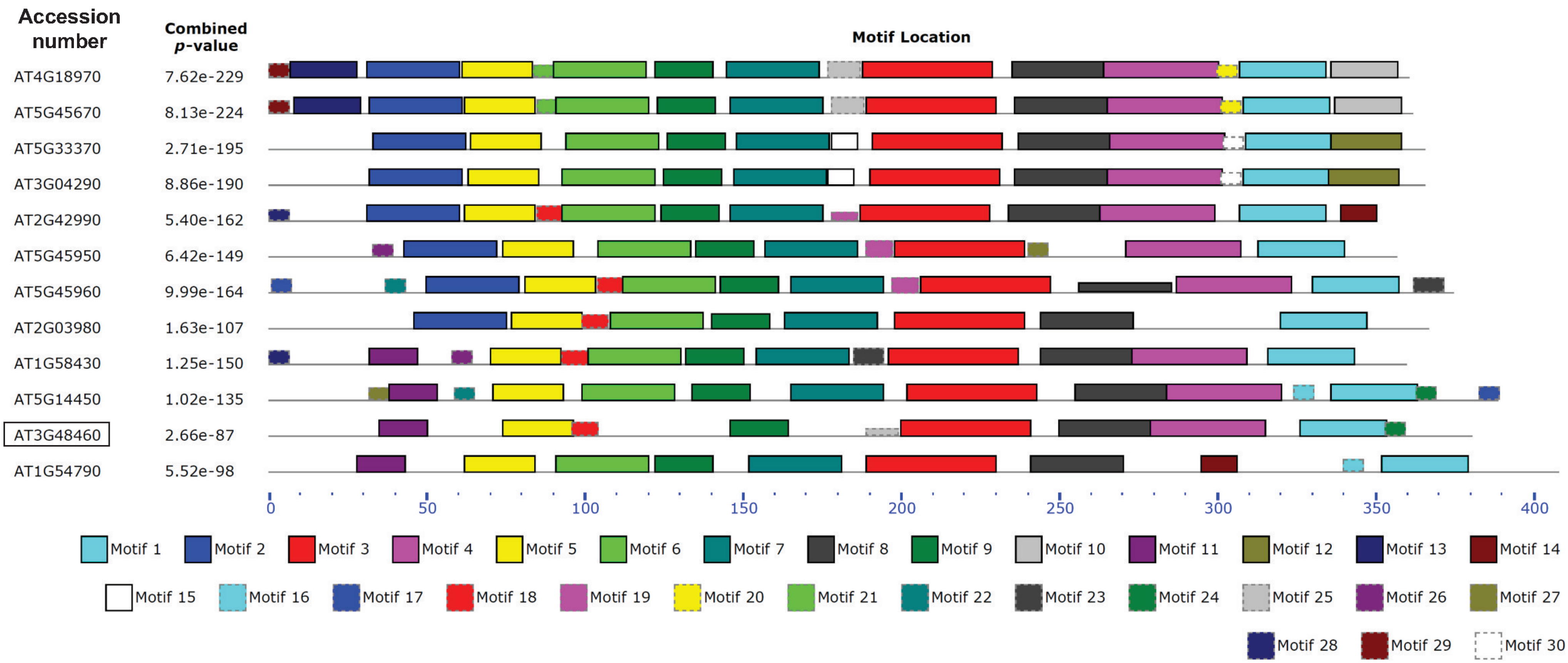

Supplementary Figure S4. Protein motif structure and location of DELLA downregulated GDSL- type enzymes in *Arabidopsis*. Each colored box represents a particular motif. Their consensus sequence, accession numbers of DELLA regulated GDSL esterase/lipase proteins, and E-value are shown in the left frame. *At3G48460* is *SFAR4* as presented as a square box.
