# Supplementary figures and images for: Arabidopsis SFAR4 is a novel GDSL-type esterase involved in fatty acid degradation and glucose tolerance
Source: Bot Stud. 2015 Dec 1;56:33. doi: 10.1186/s40529-015-0114-6 (PMC5432905; doi:10.1186/s40529-015-0114-6)

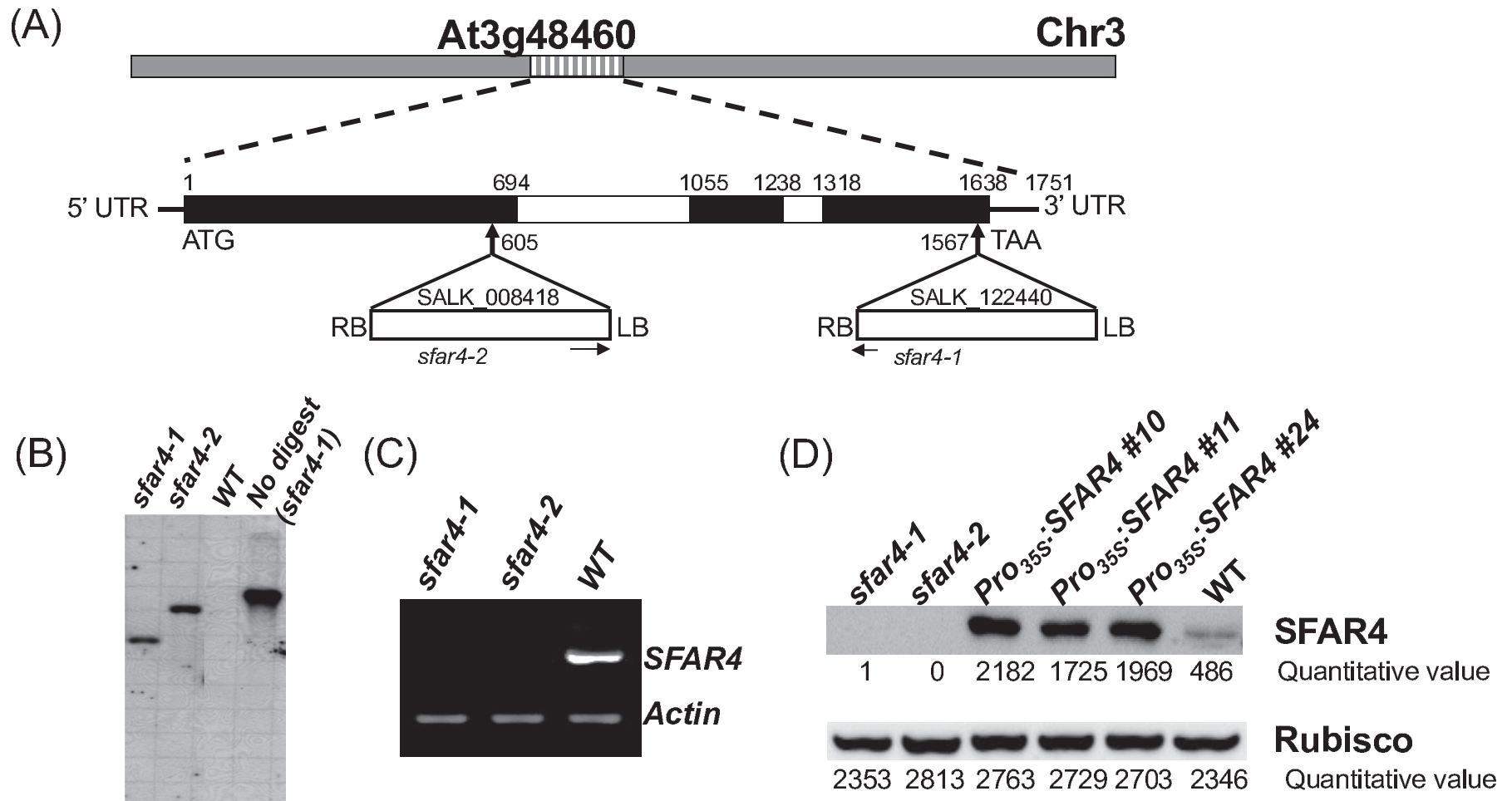

Supplement: Supplementary file 6 — Additional file 6: Figure S5. Genotype analysis of SFAR4 transgenic plants. (A) Genomic organization of SFAR4 and location of the T-DNA insertion in sfar4-1 and sfar4-2. The arrows indicate the positions of the T-DNA insertions (triangles). Genomic DNA of SFAR4 is represented by 5′UTR, exons (black), introns (white) and 3′UTR. The T-DNA orientation of the left borders (LB) is indicated by the arrow. Chr3 refers to chromosome 3. (B) Southern blot analysis of the T-DNA insertion numbers in Col-0 and sfar4 mutants. The genomic DNA was digested with HindIII, and the blot was probed with the T-DNA-specific DNA. (C) RNA analysis of SFAR4 gene expression in sfar4-1, sfar4-2, and Col-0. SFAR4 transcript expression was analyzed by RT-PCR. Total RNA (0.08 μg) was used to detect SFAR4 and Actin1 (loading control) expression. (D) SFAR4 proteins were detected by western blot with specific anti-SFAR4 antibody. RuBisCO was used as a loading control. The quantitative values were tested by Image J free software to analyze and quantify the intensity bands in western blot result images. [file 40529_2015_114_MOESM6_ESM.pdf]
