# Supplementary material for: Arabidopsis SFAR4 is a novel GDSL-type esterase involved in fatty acid degradation and glucose tolerance
Source: Bot Stud. 2015 Dec 1;56:33. doi: 10.1186/s40529-015-0114-6 (PMC5432905; doi:10.1186/s40529-015-0114-6)
Supplement: Supplementary file 7 — Additional file 7: Table S2. Accession numbers of 24 plant GDSL esterases/lipases. [file 40529_2015_114_MOESM7_ESM.docx]

**Supplementary Table 2. Accession numbers of 24 plant GDSL esterases/lipases**

| Protein name | Accession number Swiss-Prot | Properties and putative functions | Reference |
| --- | --- | --- | --- |
| AmGDSH1 (*Alopecurus myosuroides* hydrolase) | CAG27610 | Activation of aryloxyphenoxypropionate (AOPP) graminicides to bioactive herbicidal acids occurs in crops and weeds via hydrolysis of AOPP herbicide ester precursors by AmGDSH1 | (Cummins and Edwards 2004) |
| BnSCE3/BnLIP2 (*Brassica napus* sinapine esterases) | AAX59709 | Hydrolysis of sinapine during seed germination via transformation of 1-O-sinapoyl-b-glucose to sinapoyl-l-malate in the seedlings. Hydrolysis of sinapine increases nutritional value of oilseed rape seeds. | (Ling et al. 2006); Clauss et al. (2008) |
| AAE (*Rauvolfia serpentina* acetylajmalan esterase) | AAW88320 | Deacetylation of acetylnorajmaline leading to ajmaline formation (the last step in ajmaline alkaloid biosynthesis). Acetylated ajmalan alkaloids are natural substrates for AAE. | (Ruppert et al. 2005) |
| AtFXG1 (*Arabidopsis thaliana* alpha-fucosidase 1) | Q9FXE5 | Hydrolytic activity against the oligosaccharides from xyloglucan XXFG and 2′-fucosyl-lactitol of the α-l-fucosidase. | (de la Torre et al. 2002) |
| LAE (*Digitalis lanata* Ehrh. lanatoside 15′-O-acetylesterase) | CAA09694 | Deacetylation of lanatosides (one of the main type of cardenolides) to purpureaglycosides by lanatoside 15′-O- acetylesterase (LAE) catalysis. Hypothetical role of the cell-wall-bound LAE protein in cardenolide biosynthesis and transformation. | (Kandzia et al. 1998) |
| MaAChE (*Macroptilium atropurpureum* Siratro acetylcholinesterase ) | BAG09557 | The ACh-mediated system potentially localized in the extracellular region around the plasmodesmatal channel that might regulate cell-to-cell trafficking by channel gating. | (Yamamoto et al. 2008) |
| ZmAChE (*Zea mays* L. acetylcholinesterase) | Q5FC14 | Hypothetical role of the ACh-mediated system in regulation of the opening and/or closing of channels by interaction with morphoregulatory proteins at the cell wall matrix surrounding the plasmodesmata. | (Momonoki 1997) |
| AChE (*Salicornia europaea* L. acetylcholinesterase) | AB489863 | Increasing acetylcholinesterase (AChE) activity in the root and the lower part of the stem following salt (Na^+^ and Cl^-^) accumulation during growth of the Salicornia plants. | (Yamamoto et al. 2009) |
| Hev b 13 (Latex allergen *Hevea brasiliensis* 13) | Q7Y1X1 | May be involved in nodulation. Has lipase and esterase activities. | (Rouge et al. 2010) |
| ARAB-1 (Arabidopsis lipase-1) | Q38894 | Shows lipolytic activity on Tween-80 plate. | (Mikleusevic et al. 2009) |
| AgaSGNH (*Agave americana* GDSL and SGNH hydrolases) | Q5J7N0 | Extensive expression in the epidermis of the youngest leaf zones, especially active during cutin biosynthesis. Hypothetical role of an extracellular AgaSGNH protein in metabolism of the epidermis cell wall and/or in the metabolism of plant cuticle. | (Reina et al. 2007) |
| ENOD8 (*Medicago sativa* Early nodulins protein 8) | AAB41547 | The acetylesterase activity on shorter chain aliphatic ester substrates (acetyl and butyryl esters). Because of high homology to cell-wall-associated proteins, ENOD8 substrates could be acetylated oligo- or polysaccharides. | (Coque et al. 2008; Pringle and Dickstein 2004) |
| EXL4 (*Arabidopsis thaliana* pollen coat protein extracellular lipase 4) | Q0WUV7 | Promotion of the initiation of pollen coat (cuticle) hydration. | (Updegraff et al. 2009) |
| CDEF1 (*Arabidopsis thaliana* cuticle destructing factor 1) | Q9SZW7 | Facilitation of the penetration of the stigma by pollen tubes via stigma (cutin) surface destruction (hydrolysis) and the lateral roots emergence via degradation of cell-wall components. | (Takahashi et al. 2010) |
| JNP1 (*Jacaranda mimosifolia* nectar protein 1) | B0FTZ8 | Putative role in the hydrolysis of nectar lipids with the concomitant release of free fatty acids. | (Kram et al. 2008) |
| ESM1 (*Arabidopsis thaliana* epithiospecifier modifier 1) | Q9LJG3 | ESM1 represses nitrile formation and favors isothiocyanate production. | (Zhang et al. 2006) |
| MVP1 (*Arabidopsis thaliana* modiﬁed vacuole phenotype1) | Q7XA74 | Myrosinase-associated proteins play a role in myrosinase-glucosinolate metabolic plant defense system hydrolysis. | (Agee et al. 2010) |
| GLIP1 (*Arabidopsis thaliana* GDSL lipase1) | Q9FLN0 | Local and systemic acquired resistance (SAR): disruption of the spore integrity and defense signaling activation. Activation role for generation and propagation of systemic signal required for ethylene (ET)-mediated SAR. SAR: gene induced by salicylic acid (SA), jasmonic acid (JA), and ET. | (Hye Gi Kim and Cheol Na 2013; Kwon et al. 2009) |
| GLIP2 (*Arabidopsis thaliana* GDSL lipase2) | Q9SYF0 | SAR: gene induced by SA, JA and ET. Resistance to *Erwinia carotovora* via negative regulation of auxin signaling. | (Lee et al. 2009) |
| BrSIL1  (*Brassica rapa* salicylate-induced lipase-like 1 gene) | Q8L8G1 | SAR: gene is activated via SA-dependent signaling pathway in the presence of the non-host pathogen. The *Brsil1* gene is strongly induced by salicylic acid and a nonhost pathogen, *Pseudomonas syringae* pv. tomato, that elicits a hypersensitive response in Chinese cabbage. | (Lee KA 2003) |
| CaGLIP1 (*Capsicum annuum* GDSL-type lipase) | Q08ET5 | SAR: gene is activated via a SA-dependent signalling pathway, in the presence of pathogen *Xanthomonas campestris* pv. vesicatoria (Xcv). Defensive role of CaGLIP1 gene during early responses to biotic stresses. | (Hong et al. 2008) |
| CaGL1 (*Capsicum annuum* GDSL-lipase 1) | Q0R4F7 | Induction by methyl jasmonic acid (MeJA), and local/systemic wounding stimuli. Association with signalling pathway of MeJA and/or early stage of wounding responses through CaPR-4 expression modulation. | (Kim et al. 2008) |
| AtLTL1 (*Arabidopsis thaliana* Li-tolerant lipase1) | Q9M8Y5 | Induction by LiCl or NaCl and activation in the presence of SA. Halotolerance is a general function of AtLTL1 in the Arabidopsis plant. | (Naranjo et al. 2006) |
| **GLL23 (**GDSL lipase-like protein 23) | Q8W4H8 | GLL23 is normally post-translationally modified for ER export. | (Jancowski et al. 2014) |
