# Supplementary material for: Arabidopsis SFAR4 is a novel GDSL-type esterase involved in fatty acid degradation and glucose tolerance
Source: Bot Stud. 2015 Dec 1;56:33. doi: 10.1186/s40529-015-0114-6 (PMC5432905; doi:10.1186/s40529-015-0114-6)
Supplement: Supplementary file 8 — Additional file 8: Figure S6. Phenotype of SFAR4 transgenic plants. (A) Germination rates of SFAR4 transgenic plants in MS medium with or without 10mM LiCl, 150mM KCl, or 200mM NaCl. Each value is the mean of three independent measures, and the error bars correspond to the standard error (n=50). (B) Images of Col-0 and SFAR4 transgenic plants in MS medium with or without 10mM LiCl for 14 days. sfar4-1 and sfar4-2 are knockout mutants; WT, wild type; Pro 35S :SFAR4 #10 and Pro 35S :SFAR4 #11 indicate SFAR4 overexpressors, Pro 35S :SFAR4 line 10 and 11. WT is wild type (Col-0). [file 40529_2015_114_MOESM8_ESM.pdf]

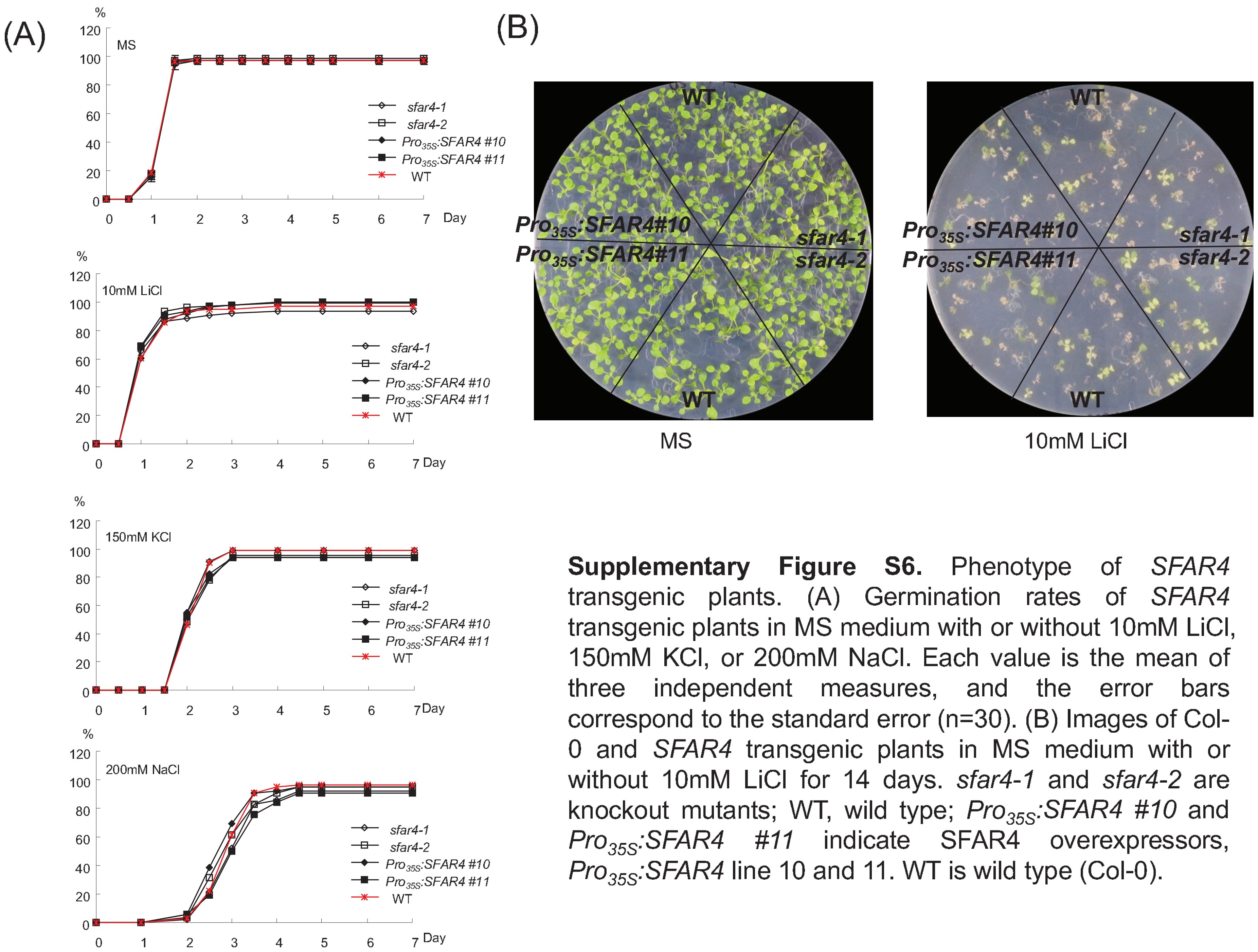

Supplementary Figure S6. Phenotype of *SFAR4* transgenic plants. (A) Germination rates of *SFAR4* transgenic plants in MS medium with or without 10mM LiCl, 150mM KCl, or 200mM NaCl. Each value is the mean of three independent measures, and the error bars correspond to the standard error (n=30). (B) Images of Col-0 and *SFAR4* transgenic plants in MS medium with or without 10mM LiCl for 14 days. *sfar4-1* and *sfar4-2* are knockout mutants; WT, wild type; *Pro*$_{35S}$*:SFAR4 #10* and *Pro*$_{35S}$*:SFAR4 #11* indicate SFAR4 overexpressors, *Pro*$_{35S}$*:SFAR4* line 10 and 11. WT is wild type (Col-0).
